# Understanding cervical cancer: an exploration of lay perceptions, beliefs and knowledge about cervical cancer among the Acholi in northern Uganda

**DOI:** 10.1186/1472-6874-14-84

**Published:** 2014-07-15

**Authors:** Amos Deogratius Mwaka, Elialilia Sarikiaeli Okello, Juliet Kiguli, Elizeus Rutebemberwa

**Affiliations:** 1 Department of Medicine, School of Medicine, College of Health sciences, Makerere University, P.O Box 7072, Kampala, Uganda; 2 Department of Psychiatry, School of Medicine, College of Health Sciences, Makerere University, Kampala, Uganda; 3 Department of Community Health and Behavioural Sciences, School of Public Health, College of Health Sciences, Makerere University, Kampala, Uganda; 4 Department of Health Policy, Planning and Management, School of Public Health, College of Health Sciences, Makerere University, Kampala, Uganda; 5 Visiting Research Scholar, Cambridge Center for Health Services Research, Institute of Public Health, University of Cambridge, Cambridge, UK

**Keywords:** Cervical cancer, Beliefs, Civil conflict, Lay explanatory model, Northern Uganda

## Abstract

**Background:**

Cervical cancer is the most common cancer affecting women in Uganda; yet community understanding of the disease is limited. We explored community perceptions, beliefs and knowledge about the local names, causes, symptoms, course, treatment, and prognosis of cervical cancer in order to inform targeted interventions to promote early help-seeking.

**Methods:**

Twenty four focus group discussions (FGD) with men and women aged 18 – 59 years and ten key informant interviews with persons aged ≥ 60 years were conducted at two sites in Gulu district between May and June 2012. A semi-structured interview guide informed by Kleinman’s illness explanatory model and literature on community awareness of cervical cancer was used to collect data. Data analysis was supported with use of ATLAS.ti 6.1 in coding, organizing and tracking data segments. We used content analysis technique in data analysis and organised data into a structured format under distinct themes and categories.

**Results:**

Cervical cancer was known by the local name “*two remo*”, meaning “an illness that manifests with bleeding.” Respondents believed that early onset of sexual activity, multiple male sexual partners and multi-parity cause cervical cancer. Respondents in half of FGDs also reported that use of condoms and family planning pills and injections cause cervical cancer. Symptoms of cervical cancer reported included vaginal bleeding, watery vaginal discharge and lower abdominal and waist pain. Respondents in most of the FGDs and key informants perceived cervical cancer as a chronic illness and that it can be treated with both modern and traditional medicines. The majority thought that cervical cancer treatment was supportive; the illness is not curable.

**Conclusions:**

While some lay beliefs about the causes of cervical cancer suggest some understanding of aetiology of the disease, other perceived causes particularly those related to use of family planning and condoms are potentially hurtful to public health. Awareness campaigns to promote early help-seeking for cervical cancer symptoms need to be culturally-sensitive and context-specific; and include messages on symptoms, risk factors, course, treatment and prognoses.

## Background

Cervical cancer is the third most common cancer and fourth leading cause of cancer death in women worldwide. Up to 83% of the new cervical cancer cases and 85% of deaths from the cancer occur in the low- and middle-income countries (LMICs) [1,2]. Most cervical cancer patients in the LMICs are diagnosed in late stages [3-7]. The reasons for late presentations are not well understood. Socio-cultural and patient-related beliefs, factors related to health systems and the biological nature of the disease might all contribute to delayed help-seeking and late stage cervical cancer at diagnosis. A history of lack of screening and tumour differentiation status were found to be statistically significantly related with late stage cervical cancer at presentation in Zimbabwe [7] while in India, socioeconomic and other patient-related factors were found to greatly influence cervical cancer stage at presentation [8]. In a study in South Africa, delay by health professionals was perceived as responsible for late diagnosis [9].

The objective of this study was to explore lay perceptions, beliefs and knowledge about cervical cancer local names, causes, symptoms, course, treatment, and prognosis so as to inform interventions to promote early biomedical help-seeking for symptoms suggestive of cervical cancer.

## Methods

### Theoretical framework

We investigated perceptions, beliefs and knowledge that may guide help-seeking for cervical cancer symptoms in the study community. Help-seeking is influenced by people’s illness representations which originate from past experiences with illness, bodily sensations, and information gathered from social networks [10-12]. Illness representations are not static and are influenced by several factors including culture and social interactions [13]. To understand illness representations, Kleinman and colleagues [14] developed illness representations models that include illness label, cause, symptoms and signs, consequences, treatment and cure. Explanatory models of ill health and misfortunes provide answers used by individuals to explain, organize, and communicate their ill health and manage particular episodes of impaired wellbeing [15]. Explanatory models are shaped by context; the explanation of an illness can be different depending on the circumstances, culture and location as well as who is giving the explanation and to whom [15]. Explanatory models provide culturally-specific explanations about illnesses, causes of distress and illnesses, and allow groups to develop shared and meaningful patterns of health promotion, health needs and care; they are natural and can be predictable [16,17].

In this study we adopted Kleinman’s explanatory model to inform the development of the interview/discussions guide and data interpretation. Lay person was operationally defined as any person who resided and worked in the study community, did not play any special roles as healers of any sort in that community and his/her work or profession was not directly related to modern medical practices. The study methods conforms to the requirement of the Qualitative research review guidelines-RATS for BioMed Central.

### Study setting

This study was conducted in Gulu district during May and June 2012. The local language in the region is Acholi/Luo [18,19]. The district is about 343 kilometres north of the Capital city Kampala and has three constituencies; Gulu Municipality, Aswa and Omoro counties. In 2011 the population of Gulu district was estimated at 396,500 people and that of the municipality at 154,300 people [20,21]. Gulu District, like neighbouring districts, experienced a prolonged civil conflict between the government of Uganda and the Lord’s Resistance Army (LRA) rebels for over two decades. The conflict which started in 1987 forced the people of Northern Uganda into congested Internally Displaced Peoples (IDP) camps where they stayed until 2006 [22].

We collected data from two villages; a village in the rural (Aswa) and a village in Municipality (urban). This was to ensure capture of any variation that may be due to geographical and socio-economic contexts of the respondents.

### Study design

A qualitative approach was adopted; Focus Group Discussions (FGD) and key informant interviews (KIIs) were used to collect data. We chose FGDs because the method could allow us generate large amounts of information in a short span of time, gain deep insights and understanding from the perspectives of the group participants on cervical cancer, and foster understanding of shared experiences and social norms that results from the social interactions and group dynamics in FGDs that would probably not emerge from a one to one interview [23-25]. We used KIIs because the method can provide deep insights that can help the researcher understand the concepts and beliefs of the community [26,27].

### Sampling

We used purposive sampling to select the two villages where we conducted this study [28]. Both villages were predominantly populated by the local (native) people who had resettled in their homesteads after the cessation of hostilities in 2006. We used purposive sampling to select respondents for the FGDs and KIIs. The key informants and members of the FGDs were identified with the help of the local council leaders of the two study villages. Respondents were invited in writing. The time and venue for FGDs were chosen to suit the participants’ convenience. The study team approached suggested key informants and made personal appointments for venues, dates and time of interviews.

### Study respondents

We conducted 24 FGDs involving 175 men and women. Each FGD had 8 – 10 members, and the groups were further stratified by age and gender (Table 1) to encourage unrestricted discussions among people of the same sex and similar age. We included community members who were born and lived in the study area, report they are Acholi, speak Acholi and were aged 18 - 59 years. We excluded from the FGDs people who self-reported and or had documented hospital diagnosis of cervical cancer, and those whose daughters or wives had a confirmed cervical cancer diagnosis because of our expectation that the experiences of people who had had a direct interface with cervical cancer would probably be influenced by the health workers’ explanatory models.

**Table 1:** Distribution of the focus groups by age groups, gender and study sites

**Gender**	**Age groups (years)**	**Rural site**	**Urban site**	**Total**
Men	18 - 29	2	2	4
	30 - 44	1	2	3
	45 - 59	2	4	6
Women	18 - 29	2	2	4
	30 - 44	2	1	3
	45 - 59	3	1	4
**Total**		**12**	**12**	**24**

Key informants (KI) were community members aged ≥ 60 years. These older people were considered knowledgeable in the culture of the local (Acholi) people and a possible influence on the transfer of beliefs and knowledge in their communities. Three men and two women respondents were interviewed in the urban site and three women and two men in the rural site. Three of the respondents (two men) were representatives in the Acholi paramount Chief’s Cultural Committee (Ker Kwaro Acholi), and were considered conversant with the culture across the whole Acholi sub-region.

### Setting of data collection

We conducted KIIs in the homes of the respondents at convenient time and in locations where no or minimal interruptions to the interview process occurred. No non-participants were allowed at interview venues.

The FGDs were conducted in convenient, quiet venues – mostly in schools, health centers or in a local government hall. The FGD respondents sat in a semi-circle, with the digital audio recorder placed in front of them. The discussion was guided by a facilitator who followed the items in the study guide and encouraged open discussions. The second research assistant managed the audio recorder, made field notes of body languages and events during discussions and interviews. In all the FGDs, the local council leaders introduced the researchers to the respondents then left the venues.

### Data collection tool

The interview/discussions guide for data collection was double translated by two independent translators, fluent in both English and Acholi/local language. Attention was paid to preservations of meaning and conceptual equivalence and consistency during translation. The translated study guide was piloted in two FGDs in a rural village (men aged 18-29 years, women aged 45-59 years) and in one urban FGD (women aged 30-44 years); and with one woman key informant. The transcripts from these FGDs and interview were reviewed against study objectives and a few probes were added in order to refine the study guide. The final tool was then developed and used in data collection in the two selected study villages.

### Data collection

We used the pre-tested/piloted semi-structured study guide for both the FGDs and KIIs. Participants were allowed to discuss uninhibited while being guided to remain focused on the topic of interest. The discussions/interviews were always started by asking respondents to name chronic illnesses they knew, especially those that affected women. This was to allow the local names of cervical cancer to emerge spontaneously. Respondents were then asked open-ended questions concerning the illness manifestations, perceived causes, course and duration, any groups at greater risk of developing the illness, problems that patients with the illness face, treatments of illness and beliefs about curability and fatality. Probes were used as necessary (e.g. Please name illnesses that affect women’s genitals and which may take long to ill? Please tell us how the illness you have described above (cervical cancer) presents itself? How does a person with the illness know they have that illness?) (See interview guide in Additional file 1). More clarifications were sought when very brief answers, ambiguous or unclear statements or very unusual ideas emerged. Interviews and discussions generally lasted between 60 and 90 minutes. We collected data until point of data saturation when no new information was coming through in further interviews/discussions [29-31]. We did not conduct repeat interviews.

### Rigour and validity

Approaches used to ensure validity included; use of experienced graduates fluent in both the local language and English as research assistants, training of the research assistants in concepts of qualitative interviewing and data collection, purpose and objectives of the current study and process of obtaining informed consents; double translation of study tool to maintain meaning and ensure reliability, conducting both the KIIs and FGDs in local language to allow for in-depth participations of the respondents who were mostly not conversant with the English language; audio recording the proceedings of the interview/discussions using a digital recorder to ensure completeness during transcriptions and translation; we performed respondents’ checking and verifications of information immediately on completing a section of the interview or discussion by paraphrasing the main aspects in the responses of the participants (members’ check); and performed triangulation of data source and data collection methods to minimize on bias and improve data richness and internal validity [32-34]. Field notes and audio-recorded information were immediately transcribed and translated into English.

### Study team and reflexivity

In order to develop a complete understanding of lay people’s perception, beliefs and knowledge about cervical cancer the first author, a native of Acholi land and who is fluent in both the Acholi and English languages, supervised the fieldwork. Data coding and analysis was done by an interdisciplinary team of clinicians and social scientists who brought in their varying expertise and experiences, and tempered any sociocultural interpretations not inherent in the data.

### Data analysis

Data analysis was carried out concurrently with data collection to allow findings to inform later data collection and determine point of data saturations. We used content analysis approach [35-37]. Analysis started with the first two authors reading through six different transcripts each. The two authors independently developed codes from the data; they thereafter discussed the codes and agreed on the appropriate codes to be used in the analysis. The first author then read through each of the transcripts line by line and applied the agreed codes to the appropriate data segments. ATLAS.ti 6.1 was used to facilitate data coding, organization and tracking changes in the dataset. The analyses proceeded with writing of memos inductively as the transcripts were read and reread. The memos were extracted and used in building themes and categories. Comparisons were made on emerging categories, subcategories and concepts both within transcripts, between transcripts and across KIIs and FGDs and rural-urban divide.

### Ethical considerations

The study protocol and consent procedures were approved by Makerere University School of Medicine Ethics Review Committee (SOMREC) and the Uganda National Council for Science and Technology (UNCST). Respondents were invited in writing. The invitation letters included purpose and objectives of the study. The process of obtaining informed consent included; introduction of research assistants, reading a consent document detailing the nature and scope of the study, purpose and objectives of the study, what is expected of respondents during data collection, anticipated benefits to community, potential risks to study participants, and confidentiality concern with data obtained during research. These were read to respondents in the local language. Prospective respondents were then allowed time to ask questions or seek clarifications, and additional explanations were provided as needed. The respondents were informed that they could withdraw their participation at any time during the course of the interview or discussions without fear of any retribution. Written informed consents were obtained from respondents. For both KII and FGDs, additional permission was obtained to audio record the interviews/discussions.

## Results

Respondents’ perceptions, beliefs and knowledge have been presented in thematic areas including cervical cancer local names, meaning of the names, cervical cancer symptoms, perceived causes, course, treatment practices and prognosis. Verbatim quotes representing consensus in FGDs and individual key informant (KI) points have been included to demonstrate particular viewpoints contributing to themes. The quotes are identified by; type of respondent (FGD or KI), gender and age or age group in brackets immediately following the quotes.

### Local names and meanings

In all the FGDs, respondents were aware of local names for cervical cancer. In all the FGDs and nine KIIs, the local name for cervical cancer given was ***“two remo”*** – an illness characterized by vaginal bleeding; a similar reference for cervical cancer was “***lutugo” -*** a descriptive term referring to the manner in which blood jets out uncontrollably from the genital of an affected woman.

### Beliefs about cervical cancer causes

In more than three quarter of the FGDs, respondents reported that cervical cancer was not common before the civil war between the government of Uganda and the LRA rebels. They blamed the foods and oils distributed to the people while in the IDP camps, the congestions in the IDPs and the fumes from bombs during the war for causing cervical cancer in Acholi land. This view cut across residence, gender and age groups.

*“I think it is the XYZ (a kind of flour) that was given to malnourished people for porridge during the camp life, because in the past before we started eating these things, these strange diseases were not there! It was not there before the LRA/NRM war in Northern Uganda! Also the cooking oil used these days which have bad smell as compared to sunflower and simsim (sesame) oil used those days are suspect” (****FGD; Urban women, aged 45 – 59)***.

In about half of both rural and urban FGDs and nine KIIs, respondents reported that cervical cancer may be caused by a virus or bacteria that is either sexually transmitted or which enters the body through other sources including poorly cooked foods.

“*When a man sleeps with an infected, bleeding woman . . . the infection the man has picked will be on his penis and when he sleeps with his wife or somebody’s wife then he transfers the infection to that woman, (****KI, Rural; man, aged 89 years****)”.*

*“There is a virus which infects you from the things you eat which are not properly cooked. This virus stays in your blood for a long time then later manifests itself in the form of the blood flow”,(****FGD, Urban; women, aged 18 – 29 years****)*.

In a few of the FGDs, respondents thought that a virus which is present in every woman right from birth is responsible for the causation of cervical cancer in some women who develop other health problems or who feed on poorly balanced diets.

*“… all people are born with viruses in their bodies. So if you do not eat well, the disease will start. So I think it starts because of poor diet” (****FGD; Urban men, 45 – 59 years)***.

Infection with syphilis was thought to cause cervical cancer when such an infection remains untreated for long or if poorly treated.

*“It is syphilis which attacks you and if left untreated, it leads to wounds in the uterus and this may not only bring barrenness but may also lead to two remo (cervical cancer),” (****FGD, Rural; women, aged 45 – 59 years).***

The majority of respondents reported that women with multiple male sexual partners are at greater risk of getting cervical cancer;

*“Multiple sexual partners especially for the lady can also cause cervical cancer because you receive ‘kwayi pig coo ma patpat’ (different kinds of sperms) in your body and this may cause a bad reaction leading to complications like the blood flow (****FGD, Rural; women, aged 18 – 29 years****).”*

In more than half of the FGDs, respondents reported that frequent and prolonged use of condoms led to cervical cancer because of adverse body reactions to the oils or lubricants on the condoms.

*“We know that the kind of oil that is used to lubricate the condoms can cause this condition (cervical cancer) especially if it causes reaction to your body and if you use it for a long time”, (****FGD, Rural; men, aged 18 – 29 years****).*

Respondents also thought that absence of menstruation, early cessation of menstruation or irregular menstrual flow were precursor states that lead to eventual accumulation of dirty blood in the woman, build-up of high pressure within the pelvis and eventual rupture of veins, thus vaginal bleeding – the tell-tale of cervical cancer.

*“When a woman stops her period early, the blood that should have been coming out with menstruation does not come out, and when the woman lives long enough the blood accumulates somewhere; it then breaks and begins to come out endlessly in large quantities” (****FGD, Rural; women, aged 30 – 44 years****).*

In almost all the FGDs, the use of family planning (FP) especially the injections was reported as a common cause of cervical cancer.

*“After a few months of using the injection family planning, blood flow starts uncontrollably or it even prevents blood flow completely. It is this ability to prevent menstruation or bring too much blood flow or even pus that can cause permanent damage leading to two remo (cervical cancer)”, (****FGD, Rural; women, Aged 45 – 59 years****).*

In addition to the causes mentioned above, some of the older women thought that cervical cancer is hereditary. A key informant (man) also reported heredity.

*“I think it is in the clan. That is why when a young woman gets the disease, the in-laws have to find out whether anyone in the family of the woman had the disease”, (****FGD, Rural; women, aged 45 – 59 years)***.

### Beliefs about cervical cancer symptoms

The majority of the key informants and respondents from all the women FGDs and about half of the men’s FGDs reported that symptoms of cervical cancer include heavy vaginal bleeding associated with very foul smelling watery discharge, lower abdominal and waist pain. However, in two urban FGDs of older men (45 – 59 years) vaginal bleeding was not considered a cardinal symptom of cervical cancer.

*“It brings a lot of pains in the waist and a watery discharge from the private parts of the woman and . . . The disease does not bring blood flow at all”,* ***(FGD, Urban; men, aged 45 – 59 years)****.*

In almost all the FGDs and key informant interviews, respondents reported that cervical cancer patients present with profound weakness which the respondents attributed to low blood levels that result from the heavy vaginal bleeding, worries about death and poor feeding due to loss of appetite.

*“The woman also becomes so weak from the loss of blood that she cannot do any work unless she is treated”, (****FGD; Rural women, aged18 - 29 years)****.*

*“You lose a lot of weight because of the worries of dying and also because of loss of appetite. The disease does not bring blood flow from the woman”,* ***(FGD; Urban men, aged 45 – 59 years)****.*

In both the FGDs and among the key informants, women were more aware of cervical cancer symptoms compared to men.

### Beliefs about cervical cancer course

In all the FGDs, respondents said that cervical cancer is a chronic illness that takes a long course, evolves over a period of time and is punctuated with changes in the nature of symptoms over time.

*“. . . generally, a woman with such disease would take about three months with the abdominal and waist pain before the flow of blood starts”,* ***(FGD, Rural; men, aged 18 – 29 years)****.*

Once bleeding has started, the majority of respondents said cervical cancer becomes chronic and can be very debilitating to the patients and increases in severity with time*.* A key informant who reported witnessing a patient suffer with cervical cancer said;

*“. . . the disease can take long in a person. The patient can take a whole year while in total pain and suffering. Within a year the patient is totally dehydrated and looks pale showing signs of having no blood in the body”,* ***(KI; Rural woman, aged 67 years)***.

### Beliefs about cervical cancer treatment and outcome

In more than half of the FGDs, respondents endorsed both traditional medicines and biomedical approaches for the treatment of cervical cancer. However, their beliefs on the potential for a cure varied by age, gender and whether they resided in rural or urban sites.

*“The medication is there both modern and traditional but most of them cannot cure. They only reduce the severity of the symptoms like blood flow and the pain”,* ***(FGD, Rural; men, aged 45 – 59 years)***.

*“There are some traditional medicines that are there . . . I saw my aunt drink it and because she took it at the beginning of the disease, it got cured. The best way to treat is through Acholi traditional medicine but not modern medicine”,* ***(FGD, Rural; women, aged 45 – 59 years)***.

*“I have an aunt who suffered from the disease and she was 60 years old. She went with it to the missionary hospital and her uterus was washed and she got cured”,* ***(FGD, Urban; men, aged 30 – 44 years)***.

In summary, respondents from both rural and urban study sites referred to cervical cancer with local names that were descriptive of the main symptom of the illness. Perceptions about causes are related to life experiences such as prolonged civil war and use of medicines such as family planning pills and injections. Respondents’ beliefs about treatments that work, and curability of cervical cancer varied by gender, age and residence in rural or urban sites.

## Discussion

The naming of an illness based on the predominant symptoms of disease, as was the case for cervical cancer, is common in Uganda. In Eastern Uganda, pulmonary tuberculosis in adults, and fever and pneumonia in children were similarly named on the basis of the main symptoms of the diseases [38-40]. Understanding the local names by which different diseases are referred to by the communities can guide the formulation of health education messages about diseases. These messages need to base on the main symptoms rather than biomedical names which in themselves may have no relevance to the community members who understand and name illnesses based on main symptoms. It is probable that illnesses that share cardinal symptoms, for example dysfunctional uterine bleeding, endometrial carcinoma and cervical cancer may be grouped together as one entity and may be treated similarly by community members. This has serious implications for treatment outcome and disease stages at presentations at biomedical facilities as these disease entities require different treatments and progress differently.

The causes of cervical cancer reported in this study included socio-culturally influenced factors including experience with civil conflict, heredity and bad luck. A recent study in Ethiopia and other earlier studies also found that knowledge of cervical cancer causes is usually mixed with beliefs which may affect treatment uptake [9,41]. The belief that cervical cancer is hereditary, for example, may lead to a failure to seek treatment in the belief that there is nothing that can be done to change a woman’s lineage. Targeted health education about the causes of cervical cancer might increase self-efficacy and perceived locus of control about cervical cancer.

We also found beliefs about causes of cervical cancer that may adversely affect other diseases prevention, public health and help-seeking practices in the community for symptoms of cervical cancer. The belief that the lubricants on condoms cause cervical cancer when condoms are used consistently for long durations may lead to non-use of condoms in spite of the proven effectiveness of condom use for control of HIV in Uganda [42,43]. Similarly, our finding that family planning injections cause cervical cancer might deter women from using this birth control method and yet Uganda has a high fertility rate (total fertility rate = 6.2 children per woman) and population growth rate of 3.2% [44]. Policymakers need to develop targeted interventions to correct misconceptions about cervical cancer.

As in this research, researchers in Laos found that knowledge of the key symptoms of cervical cancer is common among women [45]. High community awareness of cervical cancer symptoms is encouraging; women may thus recognize cervical cancer by its symptoms and might seek treatment early if they are convinced of benefits of available care at the hospitals. In the United Kingdom, awareness of cancer early symptoms was significantly associated with early help-seeking and detection [46]. Healthcare professionals need to take advantage of the high community awareness of cervical cancer symptoms to promote early help-seeking for cervical cancer.

Knowledge of the curability of cervical cancer varied by respondents’ age, gender and residence. The use of and beliefs in the potential of traditional medicines to cure cervical cancer has been reported in other countries [6,41,47]. Targeted health education on the effectiveness of biomedical modalities for treatment of cervical cancer is needed particularly in the rural communities in order to encourage early biomedical help-seeking and cautiously discourage recourse to traditional treatments for symptoms of cervical cancer.

## Conclusions

Cervical cancer is known by local names that are descriptive of the main symptoms of the disease. Lay people’s understanding of cervical cancer overlaps with biomedical knowledge of cervical cancer particularly with respect to cervical cancer symptoms, causes and treatment. The perceptions that lubricants on condoms and use of family planning injections and pills cause cervical cancer may undermine the use of these birth control methods resulting into unplanned pregnancies, abortions and maternal morbidity and mortality.

Health education messages to promote early help-seeking in biomedical facilities for cervical cancer symptoms, and private consultations need to be patient-centred and culturally sensitive; and discussion points need to cautiously and respectfully include rather than avoid any inappropriate community beliefs about cervical cancer, perceived causes and treatment modalities.

## Competing interests

The authors declare that they have no competing interests.

## Authors’ contributions

ADM conceived of the study and participated in the study design, data analysis and drafting of manuscript; ESO participated in study design, data analysis and review of manuscript; JK participated in review of manuscript for important intellectual content; ER participated in study design and review of manuscript for important intellectual content. All authors read and approved the final manuscript and consented to its submission for publication.

## Authors’ information

ADM is a clinical oncology physician and lecturer of Makerere University, and he carried out this study as part of his thesis project for the award of a PhD degree of Makerere University. ESO is a medical anthropologist and senior lecturer at the department of Psychiatry. Her PhD project was about Cultural Explanatory Model of Depression in Uganda. JK is a senior medical anthropologist and lecturer at the Department of Community Health and Behavioural Sciences at the School of Public Health, Makerere University. She has done substantial work in both medical and social anthropology. ER is an Assoc. Professor and Head, Department of Health Policy, Planning and management, at the School of Public Health, Makerere University. His PhD project was about Access to Health Care for Febrile Children in Uganda.

## Pre-publication history

The pre-publication history for this paper can be accessed here:

http://www.biomedcentral.com/1472-6874/14/84/prepub

## Supplementary Material

Additional file 1 Study Guide for Focus group discussions and key informant interviews. Click here for file
