# Supplementary material for: Understanding cervical cancer: an exploration of lay perceptions, beliefs and knowledge about cervical cancer among the Acholi in northern Uganda
Source: BMC Womens Health. 2014 Jul 15;14:84. doi: 10.1186/1472-6874-14-84 (PMC4114129; doi:10.1186/1472-6874-14-84)
Supplement: Additional file 1 — Study Guide for Focus group discussions and key informant interviews. [file 1472-6874-14-84-S1.doc]

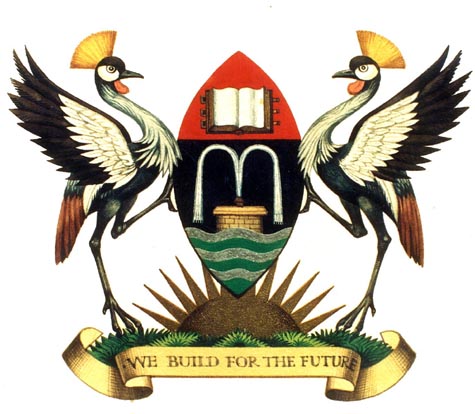

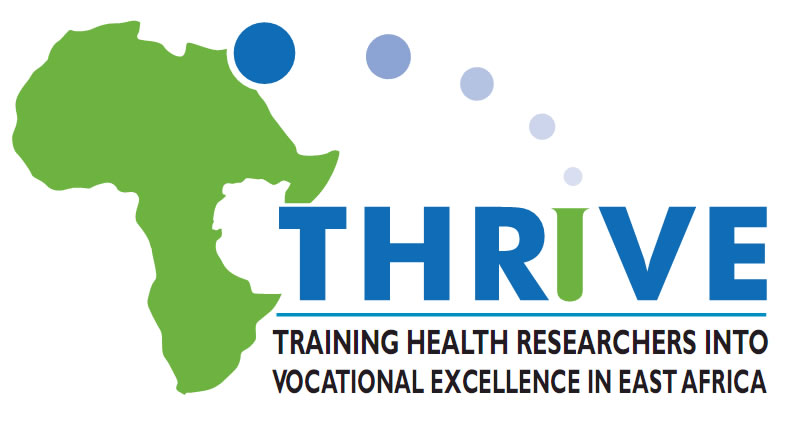
Additional file 1.

**Focus Group Discussion (FGD) and Key Informant Interview (KII) Guide**

**EXPLANATORY MODEL FOR AND ACCESS TO QUALITY BIOMEDICAL CARE FOR CERVICAL CANCER IN GULU, NORTHERN UGANDA**

**Study aim 2:** To explore beliefs, knowledge & preferred health seeking practices of Gulu community members for cervical cancer.

**Preparations:** Conduct 24 - 28 FGDs with 8 - 10 adult community members per FGD by gender and by age groups as follows: 18 – 29; 30 – 44; 45 – 59 in a rural village in Aswa County and an urban village in Gulu municipal.

**Participants:** Adult community members and elders (60+ years) of Gulu district.

**Time:** The discussion/interview is expected to last approximately 60 to 90 minutes.

**Informed consent:** Members will sign or apply thumb print after explaining purpose of study and before the discussions/interviews start. Please adhere to procedures I consent form.

**Venue:** A quiet, confidential room or open space to ensure openness and avoid interferences. No non-participant members are allowed at interview/discussions.

**Recordings:** Use the attached forms to record consent and particulars of the participants. Audio recordings will be done in addition to field notes. Obtain additional verbal consents for audio-recording.

**Introduction:** We know very little about what members of the communities in Gulu know of cervical cancer, what they think lead to or cause the illness, how the illness is managed and whether this illness can get cured or not. We know that family and community members have an understanding of what a disease/illness is and how that disease/illness affect people, how it may be managed and what to do when the disease/illness is not responding to their preferred measures of treatment. We would like to discuss with you today about aspects of cancer disease especially cancer that affect women.

**Discussion/Interview guide**

**Theme 1**. What the illness/disease is called.

**Probes**:

- What is cancer called in Acholi/Luo?

(***Do not suggest a name! Inquire about the different chronic illnesses (that take long to heal and/or feared) that they know and how they call them.***

- Specifically, what is cervical cancer called in Acholi/Luo?

***(Again, do not suggest a name. Do not describe by symptoms of the disease! Let them name the cancers i.e. “illnesses with long course” above that affect women).***

**Theme 2:** Manifestations of the illness.

**Probes:**

- What are the symptoms of the illness/cervical cancer?

***Further probes:***

- - ***Please tell us how the illness you have described above (cervical cancer) present itself.***
  - ***How does a person with cervical cancer know they have that cancer? What does a woman who has the disease (cervical cancer) feel or complain of?***
  - ***List the symptoms in the order of how commonly they present themselves among women with cervical cancer.***

**Theme 3:** Causes of the illness (cervical cancer).

**Probe:**

- Please tell us what you think or know cause cervical cancer. Allow respondent/members to enumerate freely!

***Further probes:***

- - ***After they have listed what they think cause or lead to development of cervical cancer, then inquire about smoking, sexually transmitted infections – let them name some specific infection e.g. ask if they have ever heard of HPV/some virus that causes cervical cancer.***
  - ***Inquire about multiple sexual partners, early sexual debut – if not mentioned already!***
- Please tell us about having operation done on a woman who has symptoms of cervical cancer!

***Further probes:***

- - ***Does having operation on what may be cervical cancer lead to actual development of cervical cancer?***
  - ***Does operation on cervical cancer lead to spread of the cancer to other parts of the body and lead to earlier death of the patient?***

**Theme 4:** Risk factors and groups who develop cervical cancer.

**Probes:**

- Who are the people at risk for getting cervical cancer? ***(Probe about age at developing disease, age at marriage or first sexual intercourse, occupation, smoking status, marital status/polygamy).***
- Why does the disease (cervical cancer) start in a woman?

***Further probes:***

- ***What makes the disease start?***

**Theme 5:** Problems of patients with cervical cancer.

**Probes:**

- What problems does a woman who has cervical cancer get because of the cancer being in her body?

***Further probes:***

- - ***Do people think she is less of a woman because she has a cervical cancer?***
  - ***Does she feel discriminated or stigmatized because she has cervical cancer?***

**Theme 6:** Severity, duration of cervical cancer and fatalism.

**Probes:**

- Please tell us how long cervical cancer may stay in a woman before she gets to know about it!

***Further probes:***

- - ***After how long from start of cervical cancer does the woman begin to feel symptoms of the cancer? (State weeks, months, years etc from start of cancer).***
- Can the cancer get cured?

**Theme 7:** Treatment of cervical cancer.

**Probes:**

- Please tell us how cervical cancer may be treated.

***Further probes: What medicines are used? Who gives the medicines?***

- Where do patients with cancer cervix go first for treatment? Why do they go for treatment in those places? Where do they go next and why?

**Theme 8:** Fears and concerns.

**Probes:**

- What should be done or not done (by patient, family, health workers) when a patient has cervical cancer? What is the problem with doing or not doing those things?

****Thank you for participating****
